# ChatGPT makes medicine easy to swallow: an exploratory case study on simplified radiology reports

**DOI:** 10.1007/s00330-023-10213-1

**Published:** 2023-10-05

**Authors:** Katharina Jeblick, Balthasar Schachtner, Jakob Dexl, Andreas Mittermeier, Anna Theresa Stüber, Johanna Topalis, Tobias Weber, Philipp Wesp, Bastian Oliver Sabel, Jens Ricke, Michael Ingrisch

**Affiliations:** 1 Department of Radiology, LMU University Hospital, LMU Munich, Munich, Germany; 2 Comprehensive Pneumology Center (CPC-M), Member of the German Center for Lung Research (DZL), Munich, Germany; 3 Munich Center for Machine Learning (MCML), Munich, Germany; 4 Department of Statistics, LMU Munich, Munich, Germany

**Keywords:** Natural language processing, Patient-centered care, Radiology

## Abstract

**Objectives:**

To assess the quality of simplified radiology reports generated with the large language model (LLM) ChatGPT and to discuss challenges and chances of ChatGPT-like LLMs for medical text simplification.

**Methods:**

In this exploratory case study, a radiologist created three fictitious radiology reports which we simplified by prompting ChatGPT with “Explain this medical report to a child using simple language.” In a questionnaire, we tasked 15 radiologists to rate the quality of the simplified radiology reports with respect to their factual correctness, completeness, and potential harm for patients. We used Likert scale analysis and inductive free-text categorization to assess the quality of the simplified reports.

**Results:**

Most radiologists agreed that the simplified reports were factually correct, complete, and not potentially harmful to the patient. Nevertheless, instances of incorrect statements, missed relevant medical information, and potentially harmful passages were reported.

**Conclusion:**

While we see a need for further adaption to the medical field, the initial insights of this study indicate a tremendous potential in using LLMs like ChatGPT to improve patient-centered care in radiology and other medical domains.

**Clinical relevance statement:**

Patients have started to use ChatGPT to simplify and explain their medical reports, which is expected to affect patient-doctor interaction. This phenomenon raises several opportunities and challenges for clinical routine.

**Key Points:**

• *Patients have started to use ChatGPT to simplify their medical reports, but their quality was unknown.*

• *In a questionnaire, most participating radiologists overall asserted good quality to radiology reports simplified with ChatGPT. However, they also highlighted a notable presence of errors, potentially leading patients to draw harmful conclusions.*

•* Large language models such as ChatGPT have vast potential to enhance patient-centered care in radiology and other medical domains. To realize this potential while minimizing harm, they need supervision by medical experts and adaption to the medical field.*

**Graphical Abstract:**

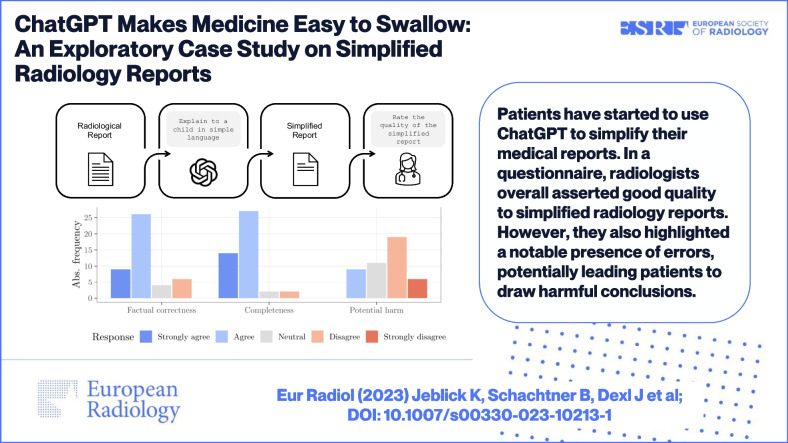

**Supplementary Information:**

The online version contains supplementary material available at 10.1007/s00330-023-10213-1.

## Introduction

“ChatGPT, what does this medical report mean? Can you explain it to me like I’m five?” With the release of OpenAI’s large language model (LLM) ChatGPT [1] on November 30th, 2022, algorithmic language modeling has reached a new milestone in generating human-like responses to user text inputs, creating potential for disruptive change across numerous domains and industries in the near future. Given the significant amount of media attention [2–5] and ever increasing level of popularity in the broad public, the question arises how people will use or even misuse such models [6], and which opportunities and challenges are associated with them.

Among a myriad of potential downstream tasks, LLMs can be applied to simplify complex text [7]. In the medical domain, there is a huge need for text simplification [8, 9]. One example is radiology: Radiological findings might have immediate consequences for patients. However, they are typically only communicated in a free-text report in specialized medical jargon, targeting a clinician or doctor as the recipient. For patients without a medical background, these reports are often inaccessible [9–12]. Offering simplified radiology reports alongside the conventional version for medical experts would allow patients to take a more active role in their own treatment process. In this context, ChatGPT and similar LLMs present unprecedented opportunities for the medical domain.

**Fig. 1:**
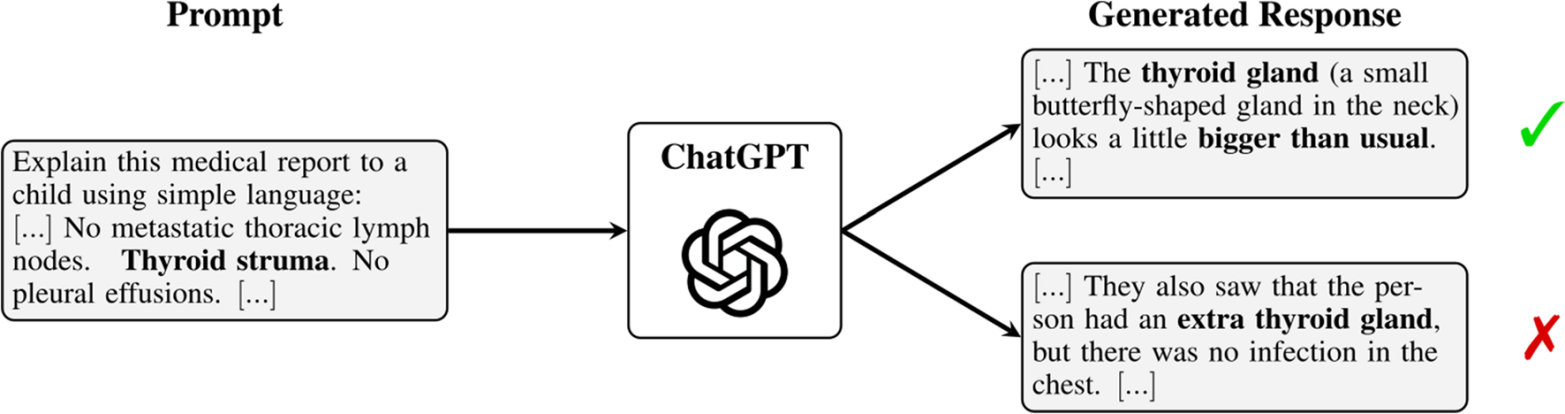
Prompting ChatGPT to simplify a radiology report of an oncological CT results in different responses each time. Even when responses sound plausible, the content does not need to be true. The response on the top correctly simplifies the term “thyroid struma,” while the response on the bottom fails to give a correct simplification

At the same time, ChatGPT was not explicitly trained for medical text simplification and is not intended to be used for this critical task. As known for other LLMs [13, 14], ChatGPT can generate plausible-sounding text, but the content does not need to be true (Fig. 1). It is even possible that a LLM adds statements that are not supported by the original text (so-called hallucinations). This raises the question of whether ChatGPT is able to simplify radiology reports such that the output is factually correct, complete, and not potentially harmful to the patient.

In this work, we conducted an exploratory case study to investigate the phenomenon that patients will autonomously utilize emerging LLMs such as ChatGPT to simplify their radiology reports. We asked 15 radiologists to rate the quality of three radiology reports simplified with ChatGPT regarding their factual correctness, completeness, and potential harm to the patient using a questionnaire. Descriptive statistics and inductive categorization were used to evaluate the questionnaire. Based on our findings, we elaborated challenges and opportunities which arise from using ChatGPT-like LLMs for simplifying radiology reports.

## Materials and methods

This prospective study is based on fictitious radiology reports, i.e., no patient information was used in this study. Written informed consent was obtained from participating radiologists. Their answers were collected in anonymized questionnaires. According to the rules of the Ethics Committee of the Faculty of Medicine, LMU Munich, no ethical consultation was therefore necessary for the approval of this study.

**Fig. 2:**
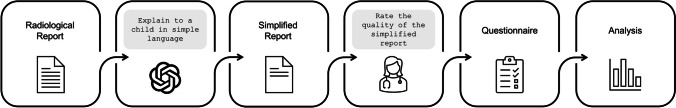
Summary of our workflow: After the creation of three fictitious radiology reports, ChatGPT is prompted to generate multiple simplified reports. Radiologists are then tasked to assess their quality in a questionnaire. Lastly, the obtained data is analyzed and evaluated

To assess the quality of radiology reports simplified with ChatGPT, we followed the workflow outlined in Fig. 2 and describe it in detail in the following.

### Original radiology reports

A radiologist with 10 years of experience wrote three fictitious radiology reports. Each report contains multiple findings, which are associated with one another, e.g., tumor with edema, meniscus lesion with cruciate ligament lesion, and systemic metastases with filiae in various body parts. All three reports are intended to be of intermediate complexity and mimic real cases in clinical routine, i.e., the reports include previous medical information, describe the findings on the image, and contain a conclusion. The first report Knee MRI (B.1.0) describes a case in musculoskeletal radiology. The case of a neuroradiological MRI of the brain is the subject of the second report Head MRI (B.2.0). It describes a follow-up examination and mentions comparisons to previous examinations. The third report (B.3.0), referred to as Oncol. CT, describes a fictitious oncological imaging event, reporting a follow-up whole-body CT scan.

### Simplification of radiology reports using ChatGPT

The original radiology reports were simplified by prompting the ChatGPT online interface [1] (version December 15th, 2022) with the request “Explain this medical report to a child using simple language:” followed by the original radiology report in plain text. This prompt was derived heuristically on separate fictitious radiology reports. Among different prompt designs, this prompt was perceived to create the best simplified reports (A.1).

The version of the interface of ChatGPT used in this study does not allow changing any model settings, resulting in non-deterministic outputs. To account for this output variability and to achieve good coverage of its generative capability, we restarted and prompted the model 15 times for each of the three original reports, respectively, i.e., we generated 15 different simplified reports per original report (Appendix B).

### Questionnaire

We designed a questionnaire (Appendix C.1) to query radiologists on the quality of the simplified reports generated with ChatGPT in the three categories (i) factual correctness, (ii) completeness, and (iii) potential harm. On the front page, the participating radiologists were informed that the simplified radiology reports were generated with “the machine learning language model ChatGPT” and received a description of the setup of the questionnaire and instructions on how to answer. Furthermore, we asked for consent to participate and the years of experience starting from the first year of residency.

Furthermore, each questionnaire contained the three original reports and, for each original report, one randomly selected, unique simplified version created with ChatGPT, followed by a series of questions to assess the quality of the simplified report (Table 1). For each quality category, participants were asked to rate their level of agreement with a corresponding statement on a 5-point Likert scale (1 = Strongly agree, 2 = Agree, 3 = Neutral, 4 = Disagree, 5 = Strongly disagree) and to additionally provide text evidence for their assessment answering a follow-up question. Fifteen radiologists with varying levels of experience from our clinic answered our questionnaire independently.

**Table 1:** Questions used to assess the quality of radiology reports simplified with ChatGPT. Likert scale statements were answered on a 5-point scale (Strongly agree to Strongly disagree), while answers to the follow-up questions were highlighted in the text in the case of factual correctness or provided as free-text responses in the cases of completeness and potential harm

Quality category	Likert scale statements	Follow-up questions
Factual correctness	“The simplified radiological report is factually correct.”	“Highlight all incorrect text passages (if applicable) of the simplified report with a text marker.”
Completeness	“Relevant medical information for the patient is included in the simplified radiological report.”	“List all relevant medical information, which is missing in the simplified report (if applicable).”
Potential harm	“The simplified report leads patients to draw wrong conclusions, which might result in physical and/or psychological harm.”	“List all potentially harmful conclusions, which might be drawn from the simplified report (if applicable).”

### Evaluation

The questionnaires were collected and checked for consent and completeness. For each participant, the years of experience were recorded. The radiologists’ ratings on the Likert scales for factual correctness, completeness, and potential harm were evaluated for each of the three cases (Knee MRI, Head MRI, Oncol. CT). Statistical parameters for the ordinal scales were calculated: median, 25%-quantile (Q_1_), 75%-quantile (Q_3_), interquartile range (IQR), minimum, maximum, mean, and standard deviation (SD). For each report, all passages of the highlighted text, as well as answers in the free-text fields, were transcribed manually to a spreadsheet (Table C. 2). Additionally, the percentage of free-text questions and text highlights where text evidence was provided by the participants was calculated. Finally, the free-text answers were inductively categorized by content.

## Results

We created for each of the three original reports 15 simplified reports (Appendix B) on the 19th to the 21st of December 2022. Even though the model is able to state to not know the answer [1], in all cases ChatGPT generated plausible-sounding outputs, while it did not indicate that the response could be incorrect. Fifteen radiologists with a median experience of 5 years (IQR [1–10]) rated the simplified reports on a 5-point Likert scale and commented on the respective follow-up questions in a free-text field. In the following, we describe the results of the Likert scale and the free-text analysis.

### Likert scale analysis

**Fig. 3:**
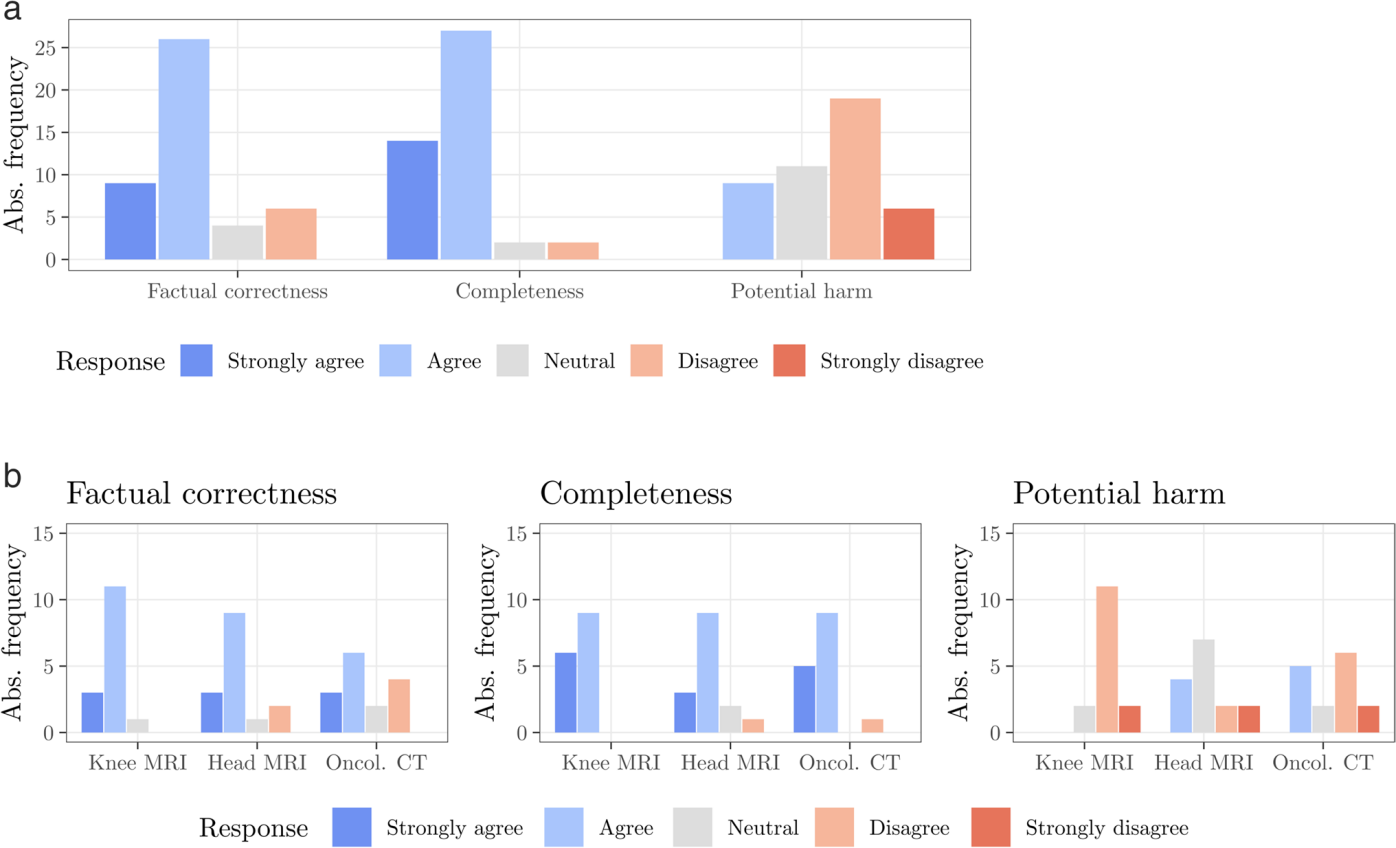
Frequency of the radiologists’ ratings for all 45 simplified reports grouped by rating category. **a** Absolute frequency of ratings for all reports combined. **b** Absolute frequency of ratings additionally grouped by original report

We first evaluated the radiologists’ ratings for all 45 simplified reports (Table 2; Fig. 3a). The participants generally agreed (median = 2) with the statements that the simplified reports are factually correct and complete, respectively. For both quality criteria, 75% of all ratings were given for “Agree” or “Strongly agree” (Q_3_ = 2), while “Strongly disagree” was not selected at all. In line with the findings for factual correctness and completeness, the participants disagreed (median = 4) on the potential of wrong conclusions drawn from the simplified reports resulting in physical and/or psychological harm. No radiologist chose “Strongly agree.”

**Table 2:** Summary statistics for the three categories factual correctness, completeness, and potential harm. Statistics for each original report in normal font and statistics for all 45 answers combined in bold font. 1 = Strongly agree, 2 = Agree, 3 = Neutral, 4 = Disagree, 5 = Strongly disagree

Question and reports	Median	Q1	Q3	IQR	Min	Max	Mean	SD
Factual correctness								
Knee MRI	2	2	2	0	1	3	1.9	0.5
Head MRI	2	2	2	0	1	4	2.1	0.9
Oncol. CT	2	2	3.5	1.5	1	4	2.5	1.1
** Combined**	**2**	**2**	**2**	**0**	**1**	**4**	**2.2**	**0.9**
Completeness								
Knee MRI	2	1	2	1	1	2	1.6	0.5
Head MRI	2	2	2	0	1	4	2.1	0.8
Oncol. CT	2	1	2	1	1	4	1.8	0.8
** Combined**	**2**	**1**	**2**	**1**	**1**	**4**	**1.8**	**0.7**
Potential harm								
Knee MRI	4	4	4	0	3	5	4	0.5
Head MRI	3	2.5	3.5	1	2	5	3.1	1
Oncol. CT	4	2	4	2	2	5	3.3	1.1
** Combined**	**4**	**3**	**4**	**1**	**2**	**5**	**3.5**	**1.0**

In the second step, we evaluated the radiologists’ ratings for each of the three cases (Knee MRI, Head MRI, and Oncol. CT) individually (Table 2; Fig. 3b). The median of the ratings for factual correctness and completeness showed no differences between the different reports. For potential harm, the Head MRI report had a slightly lower median (median = 3) compared to the other two reports (median = 4).

### Free-text analysis

Participants highlighted incorrect text passages in 23 simplified reports (51%), listed missing relevant information for 10 simplified reports (22%), and listed potentially harmful conclusions for 16 simplified reports (36%). For each follow-up question, we applied inductive categorization of the free-text data by content. We present the identified categories along with a few selected examples for each.

We first summarize our findings for the incorrect text passages. In several cases, we observed a misinterpretation of medical terms in the simplified text generated by ChatGPT. The abbreviation “DD” (differential diagnosis) was often mistaken as a final diagnosis (B.2.6, B.2.8), e.g., the simplified report contained “The conclusion of the report is that the mass on the right side of the head is a type of cancer called a glioblastoma” for the original statement “DD distant GBM manifestation.” Furthermore, “thyroid struma” was described by ChatGPT in one instance as “infection in their thyroid gland” (B.3.13) and in another as “extra thyroid gland” (B.3.7). In addition, a growing mass of “currently max. 22 mm” was incorrectly simplified as a “small, abnormal growth that has gotten bigger” (B.2.3). Some findings in the simplified reports were made up by ChatGPT. Examples for these hallucinations were “no signs of cancer in the thyroid gland” (B.3.3) or “brain does not seem to be damaged” (B.2.9). In some cases, we observed passages of imprecise language. For instance, the medial compartment of the knee was described as the “middle part of your leg” (B.1.5), or the brain as the “head” (B.2.7). Partial regression was described as “gotten smaller and is not spreading as much as before” (B.3.12). Metastases were imprecisely simplified as “spots” (B.3.5). Additionally, we found instances of unsuited and odd language. The idiom “wear and tear” (B.1.4) was used to describe the degeneration of tendons and a radiological examination was circumscribed as “to see how the cancer is doing” (B.3.8). Grammatical errors such as “CT scan” instead of “CT scanner” (B.3.7) were the exception.

The missing relevant medical information listed by the participating radiologists can be categorized as follows. We occasionally identified unmentioned findings, described in the original report but not mentioned in the simplified report. The fact that the solid portions of the pulmonary metastases are decreasing—consistent with therapy response—was not included in the simplified report B.3.5. Additionally, we observed missing or unspecific location information, e.g., imprecise localization resulted in ambiguous assignment of a growing mass to present or excised tumor (B.2.11).

Finally, we analyzed the categories from which potentially harmful conclusions might be drawn. While odd language and grammatical errors were not considered to be potentially harmful, we found examples for all other inductive categories identified above. For instance, the misinterpretation of differential diagnosis (“DD”) as final diagnosis was considered potentially harmful for the patient, e.g., one radiologist commented: “GBM is one (likely) DD which implies that other DDs exist (e.g. radionecrosis).” (B.2.7). Lymph nodes were simplified as “they might have cancer” (B.3.9), but the original report stated that there was “no evidence of recurrence or new lymph node metastases.” Furthermore, the wording “small growth” (B.2.3) was deemed a harmful conclusion, as the original report (B.2.0) states a progression in size, which “almost doubled.” For the category of hallucinations, participants rated the passage “brain does not seem to be damaged” (B.2.9) as potentially harmful to the patient, as the original report describes a growing mass. We also found potentially harmful unmentioned findings in the simplified report. The sentence “the parts that are staying the same size are changing” (B.3.5) was found to be an unclear statement missing interpretation. Missing or unspecific location of a disease can also lead to potentially harmful conclusions. For instance, radiologists listed that “Misunderstanding which lesion is stable and which one is in progress can lead the patient to some wrong expectations.” (B.2.11). Additionally, we observed that imprecise language highlighted as incorrect was sometimes also graded as potentially harmful to the patient. For example, participants listed that it is “not clear that spots are pulmonal metastases” (B.3.5) and that “some extra fluid” is not a significant increasing edema (B.2.13).

## Discussion

In this exploratory study, most participating radiologists agreed that the simplified reports were factually correct, complete, and not potentially harmful to the patient, indicating that ChatGPT is in principle able to simplify radiology reports. Nevertheless, instances of incorrect text passages and missing relevant medical information were identified in a considerable number of cases, which could lead patients to draw harmful conclusions.

In the case of incorrect text passages, we identified the inductive categories misinterpretation of medical terms, hallucinations, imprecise language, and odd language. Missing relevant medical information was categorized into unmentioned findings and unspecific location. Hallucinations are an intrinsic problem of generative models like LLMs [15] and difficult to remedy [16, 17]. The remaining inductive categories might be attributed to the fact that ChatGPT was trained on general data and not tuned to the task of radiology report simplification.

In about one-third of all simplified reports, participating radiologists found errors, which might lead patients to draw wrong conclusions, potentially resulting in physical and/or psychological harm. These errors can be grouped into the same error categories that were found for factual correctness and completeness. For example, misinterpretation of medical terms hinting at recurrence of cancer or understating progression of a disease is likely to cause harm for patients. Given that non-maleficence is a major principle of medical ethics, also in the context of AI applications [18, 19], we do see a need for further adaption of ChatGPT and similar LLMs to the medical field, in order to mitigate the risk for harmful errors. Efforts in this direction have been made, e.g., by Lee et al, Gu et al, and Singhal et al [20–22]. However, the resulting models lack the accessibility and quality of ChatGPT.

Nevertheless, ChatGPT and similar LLMs present unprecedented opportunities for text simplification in the medical domain, as their capabilities go far beyond previous machine learning approaches for medical text. So far, most authors focused on summarization of radiology reports, i.e., on the creation of a shorter version that includes all important aspects at the same level of text complexity [23–26]. In contrast, simplification does not necessarily imply shortening, but describes a transformation to make it more readable and understandable [27, 28]. Previous approaches for the simplification of radiology reports mostly replaced recurrent phrases [29–32] or augmented radiology reports with lay-language definitions [33].

Despite the positive assessment of radiologists, we see further model-related challenges when using ChatGPT for simplifying medical reports. The training data is static, i.e., new research findings are not considered in ChatGPT’s output. Also, it is well established that LLMs like ChatGPT have intrinsic biases, including stereotypical associations, or negative sentiment towards specific groups, as well as biases due to imbalanced training data [6, 34, 35]. Furthermore, ChatGPT’s output is non-deterministic, i.e., applying the same prompt multiple times results in different responses, hindering reproducibility. Finally, uploading protected health information to a proprietary service, such as ChatGPT, might compromise patients’ privacy. In summary, these model-related challenges discourage the use of ChatGPT for the task of simplification of radiology reports in an autonomous setting.

Despite these challenges, we see great potential in simplifying radiology reports with LLMs like ChatGPT to increase patients’ autonomy and facilitate patient-centered care. Simplified and thus more comprehensible reports would enable patients to better understand and oversee their own health situation and empower them to make informed and active decisions throughout the medical treatment process. Nevertheless, this should not result in patients making their own, ill-informed, clinical decisions such as delaying or even omitting further doctor appointments or terminating a therapy without professional medical consultation. We therefore envision a future integration of medical domain adapted, properly certified ChatGPT-like LLMs directly in the clinic or radiology centers. In this scenario, a simplified radiology report would always be automatically generated with an LLM based on the original report, proofread by a radiologist, and corrected where necessary. Both reports would then be issued to the patient.

This exploratory study is subject to some limitations. The number of original radiology reports (*n* = 3) and experts (*n* = 15) for assessing the quality of the simplified reports is small. The fictitious original reports were written to represent an intermediate level of medical complexity and translated to English by a non-native-speaking radiologist. We used only one phrasing of the prompt for report simplification after a heuristic selection process and did not measure the quality of simplification. In conclusion, most participating radiologists agreed that the simplified reports are overall factually correct, complete, and not potentially harmful to patients. At the same time, the radiologists also identified factually incorrect statements, missing relevant medical information, and text passages in a considerable number of simplified reports, which might lead patients to draw potentially harmful conclusions. This demonstrates the need for further model adaption to the medical field and for professional medical oversight. While further quantitative studies are needed, the initial insights of this study unveils a tremendous potential in using LLMs like ChatGPT to improve patient-centered care in radiology and other medical domains.

### Supplementary Information

Below is the link to the electronic supplementary material.

Supplementary file1 (PDF 538 KB)

## Abbreviations

IQR: Interquartile range
LLM: Large language model
Q_1_: 25%-Quantile
Q_3_: 75%-Quantile
SD: Standard deviation

## Declarations

### Guarantor

The scientific guarantors of this publication are Katharina Jeblick and Balthasar Schachtner.

### Statistics and biometry

No complex statistical methods were necessary for this paper.

### Study subjects or cohorts overlap

NA

### Methodology

• prospective

• study is based on fictitious radiology reports (exploratory study)

• performed at one institution
