# Supplementary material for: ChatGPT makes medicine easy to swallow: an exploratory case study on simplified radiology reports
Source: Eur Radiol. 2023 Oct 5;34(5):2817–25. doi: 10.1007/s00330-023-10213-1 (PMC11126432; doi:10.1007/s00330-023-10213-1)
Supplement: Supplementary file 1 — Supplementary file1 (PDF 538 KB) [file 330_2023_10213_MOESM1_ESM.pdf]

Appendix

A.1 Prompt design

The final prompt was derived in a heuristic fashion using a separate fictitious radiology report that was different from the original reports described in Appendix B. First, we tested different prompt versions of different lengths and compositions, including "Simplify", "Explain like I’m five", "Explain this medical report like I’m five", "Explain to a child using simple language", "Explain this medical report using simple language", and "Explain this medical report to a child using simple language". Based on several trials, we gained the impression that the latter prompt produced the best and most stable results. We perceived the language of the generated reports to be significantly simpler compared to the original report. We assume that this might be traced back to its detailed and explicit form, containing a concise call to action ("Explain"), specifying a narrow domain ("medical report"), and asking for a very specific level of language complexity ("to a child using simple language"). During our tests, including phrases like "to a child" or "like I’m five" in the prompt on top of "simplify" or "using simple language" appeared to be beneficial for language simplification. While our final prompt choice "Explain this medical report to a child using simple language" worked well for our radiology report examples in this study, we do not claim that this version is necessarily the best option for the task of simplifying radiology reports with ChatGPT.

B. Original and simplified reports

In Appendix B, we summarize the original reports and the simplified reports generated by ChatGPT. The incorrect text passages (question 1a) identified by participants were highlighted in the simplified reports.

B.1 Report #1: Knee MRI

B.1.0 Original Report

Radiography is used for correlation.

Extended MRI in different sequencing techniques and slice planes, application of additional, special sequencing techniques, including spectral and/or inversion-based fat suppression.

Medial compartment:

Minor cartilage damage at dorsal femoral condyle grade 2. Minor edema subchondrally, medially at medial femoral condyle DD postcontusion. Meniscus intact. Higher grade partial rupture of superficial medial ligament portions. Specifically meniscofemoral somewhat frayed deep medial ligament portions. Decent distal muscle fiber tear of the semimembranosus muscle.

Lateral compartment:

Extensive bone marrow edema at the lateral tibial plateau. No definite cartilage damage. Oblique tear at the posterior horn outer meniscus, tibial femoral side (3/8). Suspected damage to adjacent meniscopopliteal fascicles. Continuous lateral collateral ligament. If signal elevation is present, rupture of ALL suspected. Tractus iliotibialis continuous. Popliteus muscle intact.

Patellofemoral joint/extensor:

Minor tendinopathy of quadriceps tendon, tendinopathy of patellar tendon at tibial tuberosity. Patella centered. M PFL thinned out.

Intercondylar:

Anterior cruciate ligament cannot be continuously delineated, most likely complete rupture. Posterior cruciate ligament intact.

Conclusion:

- Complete rupture of the anterior cruciate ligament.

- Higher grade rupture of the medial ligament, predominantly of the superficial portions.

- Infraction at the lateral tibial plateau without step formation.

- Tear formation at the outer meniscus posterior horn. Ruptured anterolateral ligament.

Muscle fiber tear of semimembranosus muscle. Minimal cartilage damage to the medial femoral condyle.

B.1.1 Simplified Report Sample #1

Radiography is a way to take pictures of the inside of your body using special machines.

An MRI is a special kind of picture that shows more detail inside your body. It can be taken in different ways to get different types of pictures.

The medial compartment is the inside part of your leg. There is some damage to the cartilage, which is a hard, smooth material that helps your joints move easily. There is also some swelling and bruising on the inside of your knee. The meniscus, which is a c-shaped piece of cartilage in your knee, is not damaged. However, there is some damage to the ligaments, which are strong bands of tissue that help hold your bones together. There is also a small tear in a muscle called the semimembranosus muscle.

The lateral compartment is the outside part of your leg. There is a lot of swelling in the bone in the top part of your shin bone. There is no damage to the cartilage in this area. There is a tear in the meniscus, which is a c-shaped piece of cartilage in your knee, on the outside of your knee. There may also be some damage to the ligaments and muscles around your knee.

The patellofemoral joint is the joint between your knee cap (patella) and your thigh bone (femur). There is some wear and tear on the tendons that attach your thigh muscles to your knee cap and shin bone. Your knee cap is in the right position. There is some thinning of a ligament called the M PFL.

The intercondylar joint is the joint between the two rounded parts (condyles) on the top of your shin bone. The anterior cruciate ligament, which helps keep your shin bone in place, is most likely completely torn. The posterior cruciate ligament, which is on the back of your shin bone, is not damaged.

In conclusion, you have a complete tear of the anterior cruciate ligament, a higher grade tear of the medial ligament, a tear in the meniscus, and a muscle

fiber tear in the semimembranosus muscle. There is also some swelling in the bone on the outside of your shin and some wear and tear on the tendons around your knee cap. There is only minor damage to the cartilage on the inside of your thigh bone.

B.1.2 Simplified Report Sample #2

Radiography is a way of taking pictures of the inside of your body using special machines that use x-rays.

An MRI is a way of taking pictures of the inside of your body using magnets and computers. It can be used to look at different parts of your body in different ways. Sometimes, it can be used to make the pictures clearer or to highlight certain parts of your body.

The medial compartment is the inside part of your leg. There is some damage to the cartilage on the top of your shinbone and some swelling on the inside of your knee. The meniscus, which is a pad of cartilage in your knee, is okay. There is some damage to the ligaments on the inside of your knee, but they are not completely torn. There is also a small tear in one of the muscles in the back of your leg.

The lateral compartment is the outside part of your leg. There is some swelling in the bone in the top of your shinbone on the outside of your knee. There is no damage to the cartilage. There is a small tear in one of the pads of cartilage in your knee. There might be some damage to the ligaments and muscles around your knee, but it is not certain. The ligaments on the outside of your knee are okay. The muscle on the back of your leg is okay.

The patellofemoral joint is the joint between your knee cap and your thigh bone. There is some wear and tear on the tendons that attach your leg muscles to your bones. There is also some wear and tear on the tendon that attaches your knee cap to your shinbone. Your knee cap is in the right place. The ligament that holds your knee cap in place is a little thin.

The intercondylar joint is the joint between the two parts of your thigh bone. The ligament that holds your knee together in the front is most likely completely torn. The ligament that holds your knee together in the back is okay.

Overall, there is a complete tear in the ligament in the front of your knee and some tears in the ligaments and muscles on the inside and outside of your knee. There is also some damage to the bone and cartilage in your leg.

B.1.3 Simplified Report Sample #3

Radiography is a way to take pictures of the inside of your body using special machines.

The doctor used a special kind of MRI machine to take many pictures of your leg from different angles. They also used some extra techniques to make the pictures clearer.

In the middle part of your leg, there is a little bit of damage to the cartilage (a tough, rubbery substance that helps your joints move smoothly) at the top of your shin bone. There is also some swelling and bruising around the joint at the top of your shin bone. The cartilage in the middle of your knee is not damaged. The meniscus (a cushion in your knee) is not damaged either. However, some of the ligaments (tough, fibrous bands of tissue that hold your joints together) on the inside of your knee are slightly torn or frayed. There is also a small tear in a muscle called the semimembranosus muscle.

On the outside of your leg, there is a lot of swelling in the bone around the joint at the top of your shin bone. The cartilage in this area is not damaged. There is a tear in the meniscus (a cushion in your knee) on the outside of your knee. It is possible that some other tissues around the meniscus are also damaged. The ligament on the outside of your knee (called the lateral collateral ligament) is not torn. However, there may be a tear in another ligament called the anterolateral ligament. The muscle called the popliteus is not damaged.
In the front of your knee, the tendons (tough, fibrous cords that connect muscles to bones) that help straighten your leg are a little bit sore. The patella (your kneecap) is in the right place. There is some thinning of a ligament called the M PFL.

In the back of your knee, the ligament that helps keep your shin bone in place (called the anterior cruciate ligament) is most likely completely torn. The ligament that helps keep your thigh bone in place (called the posterior cruciate ligament) is not damaged.

Overall, it looks like you have a complete tear in the anterior cruciate ligament and higher grade tears in the medial ligaments in your knee. There is also a tear

in the meniscus on the outside of your knee and a possible tear in the anterolateral ligament. There is a small tear in the semimembranosus muscle and minimal cartilage damage in the medial femoral condyle.

B.1.4 Simplified Report Sample #4

Radiography is a type of medical test that uses special machines to take pictures of the inside of your body.

An MRI is another type of medical test that uses strong magnets and radio waves to take detailed pictures of the inside of your body. Sometimes, doctors will use different techniques or change the way the MRI machine takes pictures to get a better look at certain areas. They might also use special techniques to help them see certain tissues more clearly.

The medical report is talking about a person's knee. The knee has two compartments, the medial compartment (the inner part) and the lateral compartment (the outer part).

In the medial compartment, there is some minor damage to the cartilage (a smooth, rubbery material that covers the bones and helps them move smoothly against each other). There is also some swelling and minor damage to the bone in this area. The meniscus (a c-shaped piece of cartilage that acts like a shock absorber) is okay, but there is some damage to the ligaments (tough bands of tissue that hold the bones together). There is also a small tear in the semimembranosus muscle (a muscle in the leg).

In the lateral compartment, there is some swelling in the bone and no definite cartilage damage. There is also a tear in the meniscus and possible damage to other tissues. The lateral collateral ligament (another ligament in the knee) is okay. There is a possibility of damage to the anterolateral ligament (another ligament in the knee), and the popliteus muscle (another muscle in the leg) is okay.

In the patellofemoral joint (the joint between the patella, or kneecap, and the femur, or thigh bone) and the quadriceps tendon (a tendon that connects the quadriceps muscle to the patella), there is some minor wear and tear. The patella (kneecap) is in the correct position. The medial patellofemoral ligament (a ligament in the knee) is thinned out.

In the intercondylar area (the area between the two bones that make up the lower part of the leg), the anterior cruciate ligament (a ligament in the knee) is most likely completely ruptured (broken). The posterior cruciate ligament (another ligament in the knee) is okay.

The conclusion of the medical report is that the person has a complete rupture of the anterior cruciate ligament, higher grade rupture of the medial ligament (mostly the superficial portions), a small fracture in the lateral tibial plateau without a step formation, a tear in the outer meniscus posterior horn, and a muscle fiber tear of the semimembranosus muscle. There is also minimal cartilage damage to the medial femoral condyle.

B.1.5 Simplified Report Sample #5

Radiography is a way doctors can take pictures of the inside of your body using special machines.

The doctors used a special machine called an MRI to take detailed pictures of your legs. They looked at different parts of your legs and used different techniques to get the best pictures.

In the middle part of your leg (called the medial compartment), there was a small amount of damage to some cartilage on your knee and a small amount of swelling. The meniscus, which is a small, crescent-shaped piece of cartilage in your knee, was not damaged. However, some of the ligaments that help hold your knee together were partially torn. There was also a small tear in a muscle called the semimembranosus muscle.

In the outer part of your leg (called the lateral compartment), there was a lot of swelling in the bone in your lower leg and no damage to the cartilage. There was a tear in the meniscus in your knee on the outer side. There might also be some damage to some other ligaments and tissues near the meniscus. The lateral collateral ligament was not damaged, but there might be a problem with the anterolateral ligament. The muscle called the popliteus was not damaged.

In the front of your knee (called the patellofemoral joint), there was a small amount of wear and tear on the tendons that connect your thigh muscle to your knee and shin bone. The patella, or kneecap, was in the right place. There was also some thinning of the ligament called the M PFL.

In the area between the bones of your knee (called the intercondylar area), the anterior cruciate ligament was most likely completely torn. The posterior cruciate ligament was not damaged.

Overall, the main problems were a complete tear in the anterior cruciate ligament, a higher grade tear in the medial ligament, a tear in the meniscus in the outer part of the knee, a small tear in a muscle in the middle part of the leg,

and some swelling in the bone in the outer part of the leg. There was only a small amount of damage to the cartilage in the middle part of the leg.

B.1.6 Simplified Report Sample #6

Radiography is a test that uses X-rays to take pictures of the inside of the body. The doctors used this test to get more information about the patient's injuries.

The MRI is a test that uses strong magnets and radio waves to take detailed pictures of the inside of the body. The doctors used different techniques and took pictures from different angles to get a better look at the patient's injuries.

The patient had some injuries to their knee. There was some damage to the cartilage (a hard, smooth surface that covers the ends of bones) on the top part of the shinbone. There was also some swelling and injury to the cartilage on the inside of the knee. The meniscus (a cushion in the knee) was not damaged. However, some of the ligaments (tough bands of tissue that hold bones together) on the inside of the knee were partly torn. The patient also had a small tear in a muscle called the semimembranosus muscle.

On the outside of the knee, there was swelling in the bone and some damage to the meniscus. There might also be damage to other tissues in the knee. The ligaments on the outside of the knee were not torn. The popliteus muscle (a muscle in the back of the knee) was not damaged.

The patient also had some injuries to the tendons (tough cords of tissue that connect muscles to bones) in their knee. The quadriceps tendon (a tendon in the front of the thigh) and the patellar tendon (a tendon in the front of the shinbone) were slightly damaged. The patella (kneecap) was in the correct position. The ligament that helps hold the patella in place was slightly damaged.

In the middle part of the knee, the anterior cruciate ligament (a ligament that helps keep the knee stable) was completely torn. The posterior cruciate ligament (another ligament that helps keep the knee stable) was not damaged.

In summary, the patient had a complete tear of the anterior cruciate ligament and partial tears of the ligaments on the inside of the knee. There was also some damage to the cartilage on the inside of the knee, swelling in the bone on the outside of the knee, and a tear in the meniscus on the outside of the knee. The patient also had some slight damage to the tendons in the front of the leg and the patella.

B.1.7 Simplified Report Sample #7

Okay, let's break this down into simpler language.

Radiography is a type of medical test that uses special machines to take pictures of the inside of your body. This can help doctors understand what might be wrong and how to help you.

An MRI is another type of medical test that also takes pictures of the inside of your body. It uses magnets and radio waves instead of x-rays to get these pictures. Sometimes, doctors need to do a special type of MRI where they take even more detailed pictures from different angles, or they use extra techniques to get a clearer image.

Your knee has different parts, or compartments, that can be injured. The medial compartment is the inside part of your knee, and the lateral compartment is the outside part.

In the medial compartment, there is some minor damage to the cartilage on the top of your thigh bone. Cartilage is a tough, slippery material that helps your bones move smoothly against each other. There is also some swelling and a little bit of injury to the cartilage on the inside part of your knee. The meniscus, which is a C-shaped piece of cartilage in your knee, is not damaged. However, there is some damage to the ligaments, which are strong bands of tissue that hold your bones together and help your knee move. There is also a small tear in one of the muscles in your thigh.

In the lateral compartment, there is a lot of swelling and injury in the bone inside your lower leg bone. There is no damage to the cartilage in this area. There is a tear in the meniscus, which is the C-shaped piece of cartilage in your knee, on the outside part of your knee. There might also be some damage to other tissues in this area. The ligament on the outside of your knee is not damaged. There is also no damage to the muscle in the back of your knee.

In the patellofemoral joint, which is the joint where your kneecap (patella) and thigh bone (femur) meet, there is some minor damage to the tendons, which are strong cords of tissue that attach muscles to bones. The kneecap is in the right place. There is also some thinning of the ligament that helps hold the kneecap in place.

In the intercondylar area, which is the area between the two bumpy parts (condyles) at the top of your thigh bone, the anterior cruciate ligament (ACL) is completely torn. The ACL is a very important ligament that helps keep your knee stable. The posterior cruciate ligament (PCL), which is another important ligament in your knee, is not damaged.

Overall, this report is saying that you have a complete tear of the ACL and some other injuries to the ligaments, bones, and cartilage in your knee. You also have a small tear in a muscle in your thigh and some minor damage to the tendons around your kneecap. There is minimal damage to the cartilage on the inside part of your knee.

B.1.8 Simplified Report Sample #8

Radiography is a way to take pictures of the inside of your body using special equipment. MRI stands for magnetic resonance imaging. It's a way to get even more detailed pictures of the inside of your body by using magnets and radio waves. Different techniques and slice planes are just different ways to take the pictures.

The medial compartment is the inner part of your knee. There is some damage to the cartilage, which is the smooth layer on top of the bone that helps your knee move smoothly. There is also some swelling and bruising on the inner part of the bone. The meniscus, which is a cushion in your knee, is okay. But there is some damage to the ligaments, which are strong bands of tissue that hold your knee together. There is also a small tear in one of the muscles that helps you bend your leg.

The lateral compartment is the outer part of your knee. There is a lot of swelling in the bone and some damage to the meniscus, which is the cushion in your knee. There is also possible damage to other tissues in your knee. The lateral collateral ligament, which is another strong band of tissue that helps keep your knee stable, is okay. The iliotibial band and the popliteus muscle, which are other tissues that help your leg move, are also okay.

The patellofemoral joint is the joint where your kneecap (patella) moves against your thigh bone (femur). There is some minor irritation of the tendons, which are strong cords of tissue that connect your muscles to your bones. The kneecap is in the right position. The medial patellofemoral ligament, which is a strong band of tissue that helps keep your kneecap in place, is thinned out.

The intercondylar area is the space between the bones (condyles) in your knee. The anterior cruciate ligament, which helps keep your knee stable when you move, is most likely completely torn. The posterior cruciate ligament, which is another ligament that helps keep your knee stable, is okay.

Overall, there is a complete tear of the anterior cruciate ligament and some other damage to the ligaments and muscles in your knee. There is also some swelling in the bone and minor damage to the cartilage.

B.1.9 Simplified Report Sample #9

Radiography is a medical test that takes pictures of the inside of your body. It helps doctors understand what is happening inside your body.

An MRI is another medical test that takes pictures of the inside of your body. It uses strong magnets and radio waves to create detailed pictures of your bones and tissues. The MRI in this report used different ways of taking pictures and looked at different parts of the body.

The report mentions several parts of the body that were examined, including the medial compartment, which is the inside part of the knee, and the lateral compartment, which is the outside part of the knee. The report also mentions the patellofemoral joint, which is the joint between the kneecap and the thigh bone, and the intercondylar, which is the area between the two bones that make up the lower part of the leg.

The report says that there is some damage to the cartilage, which is a hard, smooth substance that helps protect the bones, in the medial compartment. There is also swelling and some damage to the ligaments, which are strong bands of tissue that hold the bones together, in the medial compartment. The meniscus, which is a crescent-shaped piece of cartilage in the knee, is not damaged. However, there is a tear in the muscle called the semimembranosus muscle.

In the lateral compartment, there is swelling in the bone and a tear in the meniscus. There may also be damage to the ligaments and muscle called the iliotibialis. The popliteus muscle is not damaged.

In the patellofemoral joint and extensor, there is some wear and tear on the tendons, which are strong cords of tissue that connect muscles to bones, and the patella, which is the kneecap, is in the correct position.

In the intercondylar, the anterior cruciate ligament, which is a ligament in the knee, is most likely completely torn. The posterior cruciate ligament, which is another ligament in the knee, is not damaged.

Overall, the report says that there is a complete tear in the anterior cruciate ligament and some damage to other ligaments and muscles in the knee. There is also some swelling in the bone and a tear in the meniscus. However, there is minimal damage to the cartilage in the medial compartment.

B.1.10 Simplified Report Sample #10

Radiography is a special kind of test that uses x-rays to create pictures of the inside of your body. The doctors used this test to help them understand what is happening in your body.

The MRI is another special test that uses magnets and computers to create detailed pictures of the inside of your body. The doctors used different techniques and planes to get a good look at different parts of your body.

In the medial compartment (the inside part) of your leg, there is some minor damage to the cartilage (the smooth, white material that covers the ends of bones) on the top part of your thigh bone. There is also some swelling and minor damage to the cartilage on the inside part of the bone at the bottom of your thigh. The meniscus (the c-shaped piece of cartilage in your knee) is okay. However, there is some damage to the ligaments (the strong, elastic bands of tissue that hold bones together) on the inside of your leg. There is also some damage to the muscle (the tissues in your body that help you move) on the back part of your thigh.

In the lateral compartment (the outside part) of your leg, there is some swelling in the bone marrow (the spongy tissue inside your bones that helps make blood cells) on the outer part of your shin bone. There is no damage to the cartilage in this area. However, there is a small tear in the meniscus (the c-shaped piece of cartilage in your knee) on the outer part of your leg. There might also be some damage to the tissues around the meniscus. The ligament (the strong, elastic bands of tissue that hold bones together) on the outside of your leg is okay. The muscle (the tissues in your body that help you move) on the back of your leg is also okay.

In the patellofemoral joint (the joint where your knee cap meets your thigh bone) and the extensor muscles (the muscles that help you straighten your leg), there is some minor damage to the tendons (the tough, white cords that connect your muscles to your bones) in your quadriceps (the group of muscles at the front of your thigh) and your knee cap. Your knee cap is in the right position. The ligament (the strong, elastic bands of tissue that hold bones together) on the inside of your leg is thinned out.

In the intercondylar (the space between the two parts of your thigh bone) area, the anterior cruciate ligament (ACL, one of the main ligaments in your knee) is probably completely torn. The posterior cruciate ligament (PCL, another main ligament in your knee) is okay.

In conclusion, you have a complete tear of the ACL ligament and some higher grade tears of the ligaments on the inside of your leg. There is also some damage to the bone on the outer part of your shin and a tear in the meniscus on the outer part of your leg. You have a small tear in the muscle on the back of

your thigh and some minor damage to the cartilage on the inside part of the bone at the bottom of your thigh.

B.1.11 Simplified Report Sample #11

This medical report is talking about a person's knee. The knee is made up of different parts, including the thigh bone, shin bone, and kneecap. The report says that there are some problems with the knee.

One problem is that there is a tear in the ligament, which is a strong band of tissue that connects bones. The ligament on the inside of the knee has a higher grade tear, which means it is more serious. There is also a tear in a muscle called the semimembranosus muscle.

Another problem is that the knee has some swelling, which is called edema. There is also some damage to the cartilage, which is a smooth, white tissue that covers the ends of the bones and helps them move smoothly.

There is also a problem with the knee joint on the side of the knee. There is swelling in the bone and a tear in the meniscus, which is a C-shaped piece of cartilage that helps cushion the joint. There may also be a problem with the ligament on the side of the knee.

Finally, the report says that there may be a problem with the ligaments that connect the thigh bone to the shin bone. One of these ligaments, called the anterior cruciate ligament, may be completely torn. However, the other ligament, called the posterior cruciate ligament, is still okay.

Overall, this medical report is saying that the person has some injuries to their knee that will need to be treated.

B.1.12 Simplified Report Sample #12

Radiography is a type of medical test that helps doctors see inside your body using special machines. They use it to help them understand what might be causing a problem or injury.

An MRI is another type of medical test that uses powerful magnets and radio waves to take pictures of the inside of your body. There are different ways the doctors can use the MRI machine to get different types of pictures. They might also use other special techniques to get even clearer pictures.

The medial compartment is the inside part of your leg. There is some damage to a small piece of cartilage on the top part of the bone in your leg. There is also some swelling and a little bit of injury to the bone under the cartilage. The meniscus, which is a small, C-shaped piece of cartilage in your knee, is not injured. But some of the ligaments that help hold your knee together are a little bit damaged or frayed. There is also a small tear in one of the muscles in your leg.

The lateral compartment is the outside part of your leg. There is some swelling in the bone in the top part of your leg. There is no damage to the cartilage in your knee. There is a small tear in a part of the meniscus that is on the outside of your leg. There might also be some damage to other small ligaments near the meniscus. The ligament that helps hold your leg and knee together is not damaged. The muscle that helps you bend your leg is also not damaged.

The patellofemoral joint is the joint between your kneecap and your leg bone. There is some minor injury to the tendons in your leg that help you straighten your leg. There is also some injury to the tendon that goes from your kneecap to your leg bone. Your kneecap is in the right position. There is some thinning of a ligament that helps hold your kneecap in place.

The intercondylar is the area between the two bones in your leg. The ligament that helps hold your leg bones together in the front is most likely completely torn. The ligament that helps hold your leg bones together in the back is not damaged.

The conclusion is that you have a complete tear in the front ligament in your knee, a higher grade tear in some of the ligaments on the inside of your leg, some swelling in the bone on the outside of your leg without any step formation, a tear in the meniscus on the outside of your leg, and a small tear in one of the muscles in your leg. There is only minimal damage to the cartilage on the inside of your leg bone.

B.1.13 Simplified Report Sample #13

Radiography is a way to take pictures inside the body using special machines. The pictures help doctors see what is going on inside the body.

MRI is another way to take pictures inside the body. It uses strong magnets and radio waves to make the pictures. Sometimes, doctors need to use different techniques or planes to get the best pictures.

The medial compartment is the part of the knee on the inside. In this case, there is some damage to the cartilage, which is the smooth white material that helps the bones move smoothly. There is also some swelling and bruising on the bone under the cartilage. The meniscus, which is a c-shaped piece of cartilage in the knee, is okay. But some of the ligaments, which are strong bands of tissue that help hold the bones together, are a little bit damaged. There is also a small tear in a muscle called the semimembranosus muscle.

The lateral compartment is the part of the knee on the outside. In this case, there is a lot of swelling in the bone under the cartilage on the outside of the tibia, which is one of the bones in the leg. There is no damage to the cartilage, but there is a tear in the meniscus, which is a c-shaped piece of cartilage in the knee. There might also be damage to some of the ligaments and muscle fibers in this area. The ligaments on the outside of the knee, called the lateral collateral ligament, and the muscle called the popliteus muscle, are both okay.

The patellofemoral joint is the joint where the kneecap (patella) and thighbone (femur) meet. In this case, there is some minor damage to the tendons that attach the quadriceps muscles (which straighten the leg) and the patellar tendon (which extends the leg) to the bones. The kneecap is in the right position. The medial patellofemoral ligament (which helps hold the kneecap in place) is a little bit thin.

The intercondylar joint is the joint between the two bones (tibia and femur) in the lower leg. In this case, the anterior cruciate ligament (which helps hold the bones together and keeps the knee stable) is most likely completely torn. The posterior cruciate ligament (which also helps hold the bones together and keeps the knee stable) is still intact.

In conclusion, there is a complete tear of the anterior cruciate ligament and a higher grade tear of the medial ligament in the knee. There is also a tear in the meniscus and some damage to the lateral collateral ligament on the outside of the knee. There is a small tear in the semimembranosus muscle and minimal damage to the cartilage on the inside of the knee.

B.1.14 Simplified Report Sample #14

Radiography is a way to take pictures of the inside of your body using special machines. MRI stands for magnetic resonance imaging and is another way to take pictures of the inside of your body using magnets and radio waves. Different slices and techniques can be used to get different kinds of pictures.

The medial compartment is the part of your leg that is closest to the middle of your body. There is some minor damage to the cartilage on the top of your thigh bone, and there is some swelling near the cartilage on the top of the bone in your

lower leg. The meniscus, which is a piece of cartilage in your knee, is okay. There is also some damage to some of the ligaments on the inside of your knee. The ligaments are like strong ropes that help hold your bones together. There is also a small tear in a muscle called the semimembranosus muscle.

The lateral compartment is the part of your leg that is farther from the middle of your body. There is some swelling in the bone in the top of your lower leg, but no damage to the cartilage. There is a tear in a piece of cartilage called the meniscus on the outside of your knee. There might also be some damage to some other ligaments and muscles near your knee. The ligaments and muscles on the outside of your knee are okay.

The patellofemoral joint is the joint between your kneecap (patella) and your thigh bone (femur). There is some minor damage to the tendons, which are like strong cords that connect your muscles to your bones, in your knee. Your kneecap is in the right place. There is also some thinning of a ligament called the M PFL.

The intercondylar area is the space between the two parts of your thigh bone that stick out at the top of your knee. The anterior cruciate ligament (ACL), which is a ligament that helps hold your thigh bone and lower leg bone together, is probably completely torn. The posterior cruciate ligament (PCL), which is another ligament that helps hold your thigh bone and lower leg bone together, is okay.

In conclusion, you have a complete tear of the ACL and a higher grade tear of the medial ligament in your knee. There is also a tear in the meniscus and possibly some damage to other ligaments and muscles on the outside of your knee. There is a small tear in the semimembranosus muscle and minimal cartilage damage on the inside of your knee.

B.1.15 Simplified Report Sample #15

Sure, I can explain this medical report to a child using simple language.

Radiography is a way to take pictures of the inside of your body, usually using x-rays.

An MRI is a special machine that uses powerful magnets and radio waves to take detailed pictures of the inside of your body. It can show different parts of your body in different ways by using different techniques, like special slicing planes and special techniques to highlight or suppress certain types of tissue.

The medial compartment is the part of your body that's on the inside, closer to the center. There's some minor damage to the cartilage (a special kind of tissue that helps protect bones) on the top of your leg bone, and some swelling on the inside of your leg bone near the top. The meniscus (a special kind of tissue that helps protect your knee joint) is okay. There's also some damage to the ligaments (tissues that hold bones together) on the inside of your knee, and a small tear in the muscle (tissue that helps you move) on the back of your thigh.

The lateral compartment is the part of your body that's on the outside, farther from the center. There's some swelling in the bone tissue on the outside of your lower leg bone, but no damage to the cartilage. There's also a tear in the meniscus on the outside of your knee, and possibly some damage to nearby tissues. The ligament on the outside of your knee is okay, but there might be a tear in the ligament on the front of your thigh. The muscle on the back of your calf is okay.

The patellofemoral joint is the joint where your kneecap (patella) and your thigh bone (femur) meet. There's some minor damage to the tendons (tissues that connect muscles to bones) on the front of your thigh and your kneecap, but the kneecap is in the right place. The ligament on the inside of your knee is a little thin.

The intercondylar joint is the joint between the two parts of your thigh bone (femur). The anterior cruciate ligament (ACL) is probably completely torn. The posterior cruciate ligament (PCL) is okay.

So, the main problems are: a complete tear of the ACL, a higher grade tear of the medial ligament, a tear in the meniscus on the outside of the knee, a small tear in the muscle on the back of the thigh, and some minor damage to the cartilage on the inside of the leg bone.

B.2 Report #2: Head MRI

B.2.0 Original Report

Several previous examinations are available for comparison.

The contrast-enhancing mass on the right parietooccipital side shows a progression in size in the short-term follow-up (currently max. 22 mm, in the previous examination max. 12 mm), with significantly increasing perifocal edema. The resection area on the right frontal side remains essentially unchanged, and there is still no definite evidence of a new contrast-enhancing

mass. Otherwise, no evidence of recent or intermediate ischemia. No evidence of intracranial hemorrhage. No signs of cerebrospinal fluid circulation disturbance.

Conclusion:

- In the short-term follow-up, significantly size-progressive contrast-enhancing space-occupying lesion right parietooccipital with significantly increasing perifocal edema, DD distant GBM manifestation.

No evidence of a contrast-enhancing mass in the right frontal resection area.

B.2.1 Simplified Report Sample #1

This medical report is talking about a person's brain. There have been some previous exams that doctors can compare to the current one.

There is a mass in the right side of the brain that has gotten bigger since the last exam. It is also causing swelling around it. There is no new mass in the right side of the brain, but there is no evidence of any problems with the blood vessels or bleeding in the brain. There are also no problems with the fluid that surrounds the brain.

The doctors think that the mass in the right side of the brain is likely a type of brain cancer called glioblastoma.

B.2.2 Simplified Report Sample #2

This medical report is talking about a person's brain. There have been previous times when doctors have looked at the brain and taken pictures of it. The report says that there is a mass (a lump) on the right side of the back of the head that has grown bigger in size recently. It also says that there is swelling (edema) around the mass. The report also mentions that there was an area on the right side of the front of the head that the doctors removed something from before, and there is no new mass there now. The report also says that there is no evidence of bleeding in the brain or any problems with the fluid that surrounds the brain. The conclusion of the report says that the mass on the right side of the back of the head is likely a type of brain cancer called glioblastoma.

B.2.3 Simplified Report Sample #3

Okay, let's break down this medical report into simpler language.

This report is about a person who has had some medical tests done before and has had more tests done recently. The tests show that there is a small, abnormal growth in the person's brain that has gotten bigger in the short-term follow-up (recent tests). This growth is called a "mass" and it is causing swelling around it. There was no change in another area of the brain that had been treated before, and there is no new growth in that area. The tests also showed that there is no bleeding in the brain and no problems with the fluid that surrounds the brain.

The conclusion of the report is that the person has a small, abnormal growth in their brain that has gotten bigger recently and is causing swelling. This growth is called a "space-occupying lesion" and it is likely a type of brain cancer called glioblastoma (GBM). There is no evidence of a similar growth in the other area of the brain that was treated before.

B.2.4 Simplified Report Sample #4

This medical report is talking about a person's brain. The report says that there is a mass or growth in a certain area of the brain that has gotten bigger since the last time it was checked. There is also swelling around the mass. The report also says that there is no evidence of any other growths in a different area of the brain that was previously treated. The report also mentions that there is no evidence of any problems with the brain's blood flow or with the fluid that surrounds the brain. The report concludes that the mass is likely a type of brain cancer called a glioblastoma.

B.2.5 Simplified Report Sample #5

This medical report is talking about a person's brain. There are some pictures of the brain from different times, and the doctors are comparing them to see how the brain has changed.

There is a problem in the right side of the back of the brain. It is called a "mass," and it is bigger now than it was before. It is also causing some swelling around it. On the right side of the front of the brain, there is a place where the doctors took out some tissue before, and it has not changed. There is no new problem in that area.

Overall, the brain does not have any signs of not getting enough blood or oxygen, or bleeding. The fluid that surrounds the brain and spine is also normal.

The doctors think that the problem in the right side of the back of the brain might be a type of tumor called a "glioblastoma." This is a serious type of tumor that can spread to other parts of the brain.

The report concludes by saying that the problem in the right side of the back of the brain has gotten bigger and is causing swelling, and it might be a glioblastoma. There is no new problem in the right side of the front of the brain.

B.2.6 Simplified Report Sample #6

This medical report is about someone who has had a few medical exams in the past, and the doctors are comparing the results from these exams to see if anything has changed.

The report says that there is a mass (a group of abnormal cells) on the right side of the back of the head that has gotten bigger since the last exam. The mass is called a "contrast-enhancing mass" because it shows up better on certain types of medical images when a special dye is used. The mass has grown from 12 millimeters (mm) to 22 mm, which is a big change in a short amount of time. There is also swelling (edema) around the mass.

On the other side of the head, in the right frontal area, there was a surgery to remove a mass. The report says that this area looks the same as it did before and there is no evidence of a new mass forming.

The report also mentions that there is no evidence of bleeding in the brain or any problems with the fluid that surrounds the brain and spine.

The conclusion of the report is that the mass on the right side of the head is a type of cancer called a glioblastoma, and it has gotten bigger and caused more swelling in a short amount of time. There is no evidence of a new mass in the area where the surgery was done.

B.2.7 Simplified Report Sample #7

This medical report is talking about a person's brain. They have had some tests done before and are comparing them to a new test.

There is a problem in the right side of the back of the head. It is a mass (a lump) that is growing. It is also causing swelling around it.

There is no new problem in the right side of the front of the head, and there are no problems with the blood vessels or bleeding in the brain. There are also no problems with the fluid that surrounds the brain.

The doctors think that the mass on the right side of the back of the head is a type of brain cancer called a GBM.

B.2.8 Simplified Report Sample #8

This medical report is talking about a mass (a lump) that was found in the brain. This mass is growing bigger and bigger, and it is causing swelling around it. The doctors have been watching it over time, and they can see that it has gotten bigger in the short term (recently). The mass is located on the right side of the back of the head. There is no other mass in the right side of the front part of the head. The doctors do not see any problems with blood flow or bleeding in the brain, and they do not see any problems with the fluid that surrounds the brain.

The doctors think that this mass might be a type of brain tumor called a glioblastoma (GBM). It is called a "space-occupying lesion" because it takes up space in the brain and can cause problems. The doctors will continue to watch the mass and see if it gets bigger or if there are any other changes.

B.2.9 Simplified Report Sample #9

This medical report is about a person who has had some medical tests done on their brain. The report shows that there is a mass (a lump) on the right side of the brain that has gotten bigger since the last time it was checked. The mass is causing some swelling around it. There is no new mass on the right side of the head, and the person's brain does not seem to be damaged or bleeding. There is also no problem with the fluid that surrounds the brain. The doctors think that the mass might be a type of cancer called a glioblastoma.

B.2.10 Simplified Report Sample #10

This medical report is talking about a person who has had some medical tests done recently, and they also had some tests done before. The doctors are comparing the results of the new tests to the old ones to see if anything has changed.

The report says that there is a mass (a lump) on the right side of the back of the head that has gotten bigger since the last time they were checked. The mass is called a "contrast-enhancing mass" because it shows up better on the medical tests when a special dye is used. The mass is also causing some swelling around it.

The report also says that there is no new mass on the right side of the front of the head, and that there are no signs of any problems with blood flow to the brain or with bleeding in the brain. There are also no signs of any problems with the fluid that surrounds the brain.

The doctors have concluded that the mass on the back of the head is likely a type of brain tumor called a glioblastoma, and it is getting bigger. There is no new mass on the front of the head.

B.2.11 Simplified Report Sample #11

A medical report is a document that describes the results of a medical examination or test. It can be written in medical language, which is a special way of talking about the body and its functions.

This medical report is talking about a person's brain. The brain is the part of the body that controls everything we do and think.

The report says that there is a problem in the right side of the brain. It's a mass, which means it's a lump or bump. This mass is growing bigger and causing swelling around it. This is called edema.

The report also mentions that there was a surgery done on the right side of the brain before. The report says that there is no new problem in that area.

The report also says that there is no bleeding in the brain, and that the brain is getting enough blood and oxygen. It also says that the brain is not having any trouble sending messages through the body.

The conclusion of the report is that there is a growing mass on the right side of the brain that is causing swelling, and it might be a type of cancer called a GBM. But there is no new problem in the area where the surgery was done.

B.2.12 Simplified Report Sample #12

Okay, let's break this down into simpler language:

There are some past medical exams that we can compare to the current one. The current exam shows that there is a mass (a lump) on the right side of the back of the head that has grown bigger since the last exam (it's 22mm now, but it was 12mm before). There is also some swelling around the mass. There is no new mass on the right side of the front of the head, and there are no problems with blood flow or bleeding in the brain. There are also no problems with the fluid that surrounds the brain.

Overall, it looks like the mass on the right side of the back of the head has grown quickly and is causing some swelling. We think it might be a type of cancer called glioblastoma. There is no new mass on the right side of the front of the head.

B.2.13 Simplified Report Sample #13

This medical report is talking about a person's brain and some changes that have happened recently. There was a mass or growth on the right side of the brain that has gotten bigger in a short amount of time. There is also some extra fluid around this mass. On the other side of the brain, there doesn't seem to be any changes. There are no signs of problems with the blood or fluid in the brain. The doctors think that this mass might be a type of brain cancer called glioblastoma.

B.2.14 Simplified Report Sample #14

This medical report is about a person who has had some medical exams done in the past and has a new exam done recently. In the new exam, there is a mass (a

lump or growth) in the back of their head that has gotten bigger since the last exam. The area around the mass is also swollen. There was no evidence of a mass in the front of the head where the person had surgery before. The report also mentions that there is no evidence of bleeding in the brain or problems with the fluid that surrounds the brain. The doctors think that the mass in the back of the head is probably a type of brain cancer called a glioblastoma.

B.2.15 Simplified Report Sample #15

This medical report is talking about a person's brain and some changes that have happened since their last medical examination. It mentions that there is a mass or growth on the right side of the brain that has gotten bigger since the last examination. There is also some swelling around this mass. The report also mentions that there is no evidence of any other masses or growths in the brain or any problems with the blood or fluid in the brain. The conclusion of the report says that the mass and swelling on the right side of the brain are likely caused by a type of cancer called glioblastoma.

B.3 Report #3: Oncology CT

B.3.0 Original Report

Findings:
Multiple CT scans available for comparison.

Thorax:
Port system right prepectoral. Heart normal in size. No pericardial effusion. No metastatic thoracic lymph nodes. Thyroid struma. No pleural effusions. No pneumonia. No pneumothorax. Marked pleural calcifications unchanged. Pulmonary metastases show response to therapy, e.g., size regrowth of left apical metastasis at 1.9 x 1.5 cm (previously 2.2 x 2.1 cm) and decreasing solid portions with new cavitation in left subpleural metastasis in lower lobe at 1.8 cm (previously 1.9 cm). No new pulmonary metastases detected.

Abdomen:
Homogeneous contrast of liver parenchyma with no metastatic lesions. Portal vein and hepatic veins open. Gallbladder non-irritant with small calculus. No cholestasis. Spleen, pancreas and adrenal glands unremarkable. Lateral contrast of the kidneys. No urinary retention. No ascites. No free abdominal air. Z. n. resection of lymph node metastases in the mesentery, no evidence of recurrence

there. No newly metastatic abdominal lymph nodes. Unchanged prominent retroperitoneal lymph nodes, these in need of further control.

Skeleton:

No suspicious osseous lesions. No evidence of acute fractures.

Conclusion:

In metastatic colorectal carcinoma, significant response to therapy in comparison to the previous examination:

pulmonary metastases present partly in regression and partly constant, but in the latter case with decreased solid parts in the sense of a therapy response.

no evidence of recurrence or new lymph node metastases after resection of the mesenteric lymph node metastasis.

no evidence of new hematogenous distant metastases in the examination area.

B.3.1 Simplified Report Sample #1

This medical report is talking about a person who has a type of cancer called colorectal carcinoma, which means cancer that starts in the colon or rectum. The report has information about different parts of the body and how the cancer is affecting them.

The thorax is the area in the body that includes the chest and the organs inside it, like the heart and lungs. The report says that the heart is normal in size and there is no extra fluid around it. It also says that there are no infections in the lungs and no air leaking from the lungs. The report says that there are some spots on the lungs where the cancer has spread, but they are getting smaller and changing in a way that shows that the person is responding to treatment.

The abdomen is the area in the body that includes the belly and the organs inside it, like the liver, stomach, and intestines. The report says that the liver looks normal and doesn't have any cancer spreading to it. It also says that the gallbladder and spleen look normal, and there is no extra fluid in the belly. The report says that there was surgery to remove some cancer from the lymph nodes in the mesentery, which is a sheet of tissue that holds the intestines in place. The report says that there is no cancer in the lymph nodes now, but there are some other lymph nodes in the belly that need to be checked again.

The skeleton is the body's framework of bones. The report says that there are no bones with cancer and no broken bones.

The conclusion of the report is that the person's cancer is responding to treatment. It is getting smaller in some places and changing in a way that shows the treatment is working. There is no new cancer in the lymph nodes or other

parts of the body, and the surgery to remove cancer from the lymph nodes was successful.

B.3.2 Simplified Report Sample #2

This medical report is about a person who has cancer that has spread from their colon to other parts of their body. The report describes the results of some tests that were done to see how the cancer is doing.

The tests show that there are some cancer cells in the person's lungs, but they are getting smaller. There is also no evidence that the cancer has spread to other parts of the body, like the liver or bones. There were also some cancer cells removed from the person's belly, but they are not growing back.

Overall, it looks like the person is responding well to treatment, which means that the cancer is getting smaller or not spreading as much. It is important to continue to monitor the cancer and see how it responds to treatment.

B.3.3 Simplified Report Sample #3

This medical report is about a person who has cancer that has spread to other parts of their body. The report is talking about different parts of the body and whether there are any signs of cancer in those areas.

In the thorax, or chest, the report says that the person's heart is normal and there is no extra fluid around it. There are no signs of cancer in the lymph nodes in the chest or in the thyroid gland. There is no infection in the lungs and no holes in the walls between the lungs and chest. The report also mentions some spots on the person's lungs that are caused by the cancer. These spots are getting smaller, which is a good sign and means that the person is responding to treatment.

In the abdomen, or belly, the report says that the liver looks normal and there are no signs of cancer. The gallbladder and bile ducts are also normal. The spleen, pancreas, and adrenal glands are all normal. The kidneys are also normal and there is no extra fluid in the belly. The report mentions some cancer in the lymph nodes in the belly that has been removed, but there are no signs that the cancer has come back or spread to other lymph nodes.

In the skeleton, or bones, the report says that there are no signs of cancer or any recent fractures.

Overall, the report says that the person's cancer is responding to treatment. The cancer in the lungs is getting smaller and there are no new signs of cancer in the other parts of the body that were checked.

B.3.4 Simplified Report Sample #4

A medical report is a document that explains the results of different tests that were done on a person. The report has information about the person's chest (called the thorax), stomach (called the abdomen), and bones (called the skeleton).

The chest looks normal, and the heart is the right size. There is no extra fluid around the heart or in the lungs, and the person does not have an infection in the lungs. The thyroid gland is swollen, but there are no other problems in the chest. There are some spots on the lungs that are smaller than they were before and are getting better with treatment. There are no new spots on the lungs.

The stomach looks good, with no abnormal growths. The liver and gallbladder are normal, and there is no blockage in the bile ducts. The spleen, pancreas, and adrenal glands are normal. The kidneys are working well and there is no extra fluid in the stomach. There are no new abnormal growths in the lymph nodes in the stomach, and the lymph nodes in the back of the stomach will need to be checked again later.

The bones are normal and there are no broken bones.

Overall, the person's cancer is responding well to treatment. The abnormal growths in the lungs are getting smaller and there are no new abnormal growths in the lymph nodes or other parts of the body.

B.3.5 Simplified Report Sample #5

A CT scan is a special kind of X-ray that takes pictures of the inside of your body. The doctor looked at multiple CT scans from different times to see how your body has changed.

The doctor saw that your heart is normal in size and there is no extra fluid around it. The doctor also saw that your thyroid gland is bigger than normal. The thyroid is a small gland in your neck that helps control your body's functions. The doctor did not see any infection in your lungs or any air in the

space between your lungs and chest wall. There were some spots on your lungs that have gotten smaller because of the medicine you are taking. There were also some spots on your lungs that have changed, but they are not getting bigger. The doctor did not see any new spots on your lungs.

The doctor saw that your liver looks normal and there are no new spots on it. The veins that carry blood to and from your liver are open and working properly. Your gallbladder and the tube that carries bile (a substance that helps you digest food) from your liver to your intestine are normal. The spleen, pancreas, and adrenal glands (organs that help with digestion and other body functions) are normal. The doctor saw that your kidneys (organs that help filter waste from your blood) are normal and there is no urine (pee) building up in your bladder. There is no extra fluid in your belly and no extra air in your belly. The doctor saw that the lymph nodes (small organs that help fight infection) near your intestine were removed before and there are no new ones. There are some lymph nodes near your belly that the doctor will keep an eye on.

The doctor did not see any new or suspicious spots on your bones and there are no broken bones.

Overall, the doctor saw that the cancer in your body is responding to the medicine you are taking. The cancer in your lungs is partly getting smaller and partly staying the same size, but the parts that are staying the same size are changing. There is no cancer in the lymph nodes near your intestine and no new cancer in other parts of your body.

B.3.6 Simplified Report Sample #6

This medical report is about a person who has a type of cancer called colorectal carcinoma, which means the cancer started in the colon or rectum. The doctor used some special machines called CT scans to take pictures of the inside of the person's body to see how the cancer is doing.

The pictures showed that the person's heart is normal and there is no extra fluid around it. The thyroid gland (a small butterfly-shaped gland in the neck) looks a little bigger than usual, but there are no other problems in the chest. There are some spots on the person's lungs where the cancer has spread, but they are getting smaller and changing in a way that shows the person is responding to treatment. There are no new spots on the lungs.

The pictures also showed that the person's liver looks normal and there are no cancer spots on it. The gallbladder (a small organ that stores bile) has a small stone, but it's not causing any problems. The rest of the organs in the stomach area look normal too. There was some cancer in the lymph nodes (small, bean-shaped organs that help fight infections) near the stomach, but it has been

removed and there is no sign that it has come back. There are some bigger lymph nodes in the back of the stomach that the doctor will keep an eye on.

The pictures didn't show any problems with the person's bones, and there are no broken bones. Overall, the doctor thinks that the person's cancer is responding well to treatment and there is no new cancer in the body.

B.3.7 Simplified Report Sample #7

Here is a summary of the medical report in simple language:

The doctor did some special pictures of the inside of the person's body using a machine called a CT scan. They looked at the person's chest and saw that the heart was a normal size and there was no fluid around it. They also saw that the person had an extra thyroid gland, but there was no infection in the chest and no air was leaking out of the lungs. The doctor saw that the cancer in the person's lungs was getting smaller after treatment and there were no new areas of cancer.

The doctor also looked at the person's stomach and saw that the liver was healthy and there were no cancer cells there. The gallbladder was also healthy, with just a small stone inside. The other organs in the stomach, like the spleen and pancreas, were normal. The kidneys looked good and there was no extra fluid in the stomach. The doctor saw that the cancer in the person's abdomen had been removed and there was no evidence that it had come back. There were also no new areas of cancer in the abdomen.

The doctor also looked at the person's bones and saw that they were normal and not broken.

Overall, the doctor saw that the treatment for the person's cancer was working and the cancer was getting smaller. There were no new areas of cancer and no evidence that the cancer had come back.

B.3.8 Simplified Report Sample #8

The medical report is about a person who has cancer that has spread to other parts of their body. The doctors did some special pictures of the inside of the person's body to see how the cancer is doing. The cancer in the person's lungs is getting smaller in some parts and there is less solid cancer in other parts, which is good because it means the medicine the person is taking is working. The cancer has not spread to any new parts of the body and the cancer that was

removed from the person's intestine is not coming back. The bones are all healthy and there are no broken bones.

B.3.9 Simplified Report Sample #9

This medical report is talking about a person who has cancer in their colon (part of the large intestine) that has spread to other parts of their body. The doctors used a special machine called a CT scan to take pictures of the inside of the person's body to see how the cancer is doing. The CT scan showed that the cancer in the person's lungs is getting smaller in some parts and not growing as much in other parts, which is a good sign that the treatment is working. The CT scan also showed that there is no cancer in the person's liver, gallbladder, spleen, pancreas, adrenal glands, or kidneys, and that there is no cancer in the person's bones. There is also no cancer in the person's abdomen (belly) after the doctors removed some lymph nodes that had cancer in them. However, there are some lymph nodes in the person's back that the doctors need to keep an eye on because they might have cancer. Overall, the treatment seems to be helping the person's cancer get better.

B.3.10 Simplified Report Sample #10

This medical report is about a person who has cancer that has spread from the colon to other parts of the body. The report is talking about a series of tests that were done to see how the cancer is doing.

The tests show that the person's heart is a normal size and is not causing any problems. The thyroid gland is swollen, but there is no fluid or infection around the lungs. The lungs have some cancer in them, but the cancer is getting smaller and there is no new cancer. The liver and other organs in the belly are normal and there are no new cancer cells in the belly. The bones do not have any cancer and there are no new broken bones.

Overall, the cancer is responding to treatment and is not getting worse or spreading to new places. There are still some cancer cells in the lungs, but they are getting smaller. The cancer surgery in the belly was successful and there is no evidence of the cancer coming back in that area. There are no new cancer cells in any other parts of the body.

B.3.11 Simplified Report Sample #11

This medical report is about a person who has a type of cancer called colorectal carcinoma. The report mentions some scans that have been taken of the person's body.

The scans show that the person's heart is normal and there are no problems with the fluid around the heart. The thyroid gland looks swollen, but there are no signs of infection in the lungs or fluid in the space between the lungs and the chest wall. The cancer in the lungs looks like it is getting smaller and there are no new areas of cancer in the lungs.

The scans also show that the liver looks normal and there are no new areas of cancer in the abdomen. The gallbladder has a small stone, but there is no blockage of bile. The spleen, pancreas, and adrenal glands all look normal. The kidneys are normal and there is no urine build-up in the bladder. There is no fluid in the abdomen and there is no air in the abdomen. There was surgery to remove cancer in the lymph nodes in the abdomen and there is no evidence that the cancer has come back there. There are some swollen lymph nodes in the back of the abdomen that need to be watched closely.

The scans also show that there are no new or unusual areas of cancer in the bones. There are no broken bones.

Overall, the cancer in the lungs looks like it is getting smaller and there is no evidence that the cancer has spread to other parts of the body or come back after surgery.

B.3.12 Simplified Report Sample #12

This medical report is about a person who has cancer that has spread to different parts of their body. The doctors used special pictures (CT scans) to look at the person's body and check for any changes. They looked at the person's chest and saw that their heart is normal and there is no fluid around it. They also saw that the person has thyroid problems, but no fluid in their lungs or any signs of pneumonia. The cancer in the person's lungs has gotten smaller and is not spreading as much as before. The doctors also looked at the person's abdomen (stomach) and saw that their liver, gallbladder, spleen, pancreas, and kidneys are all normal. They also saw that the cancer in the person's stomach has not spread to other parts of the body and has not come back after surgery. Finally, the doctors looked at the person's bones and saw that there are no new cancer spots or broken bones. Overall, it looks like the person's cancer is responding well to treatment and is not getting worse.

B.3.13 Simplified Report Sample #13

This medical report is talking about the results of some tests that were done on a person's body. The tests included taking pictures of the inside of their body using a special machine called a CT scan. The report mentions different parts of the body, such as the thorax (the chest), the abdomen (the belly), and the skeleton (the bones).

The report says that the heart is normal in size and there is no fluid around it. It also says that the person has an infection in their thyroid gland and there is no fluid in their chest or belly. The report mentions some spots on the person's lungs that are caused by cancer. These spots have gotten smaller since the last time they were checked, which means that the treatment the person is getting is working. There are no new cancer spots on the person's lungs.

The report also mentions the person's liver, which is a big organ in the belly that helps with digestion and getting rid of waste. It says that the liver looks normal and there are no cancer spots on it. The report also mentions the person's gallbladder, spleen, pancreas, and kidneys, which are all organs in the belly that help with different functions in the body. It says that these organs look normal. The report also mentions some lymph nodes, which are small bumps that help fight infections. It says that some lymph nodes in the person's belly have been removed because they had cancer, but there is no evidence that the cancer has come back. The report also mentions the person's bones and says that there are no unusual spots on them and no broken bones.

Overall, the report says that the person has cancer that started in their colon (a part of the digestive system) and has spread to other parts of their body. The report says that the treatment the person is getting is working and there is no evidence of the cancer spreading to other parts of the body.

B.3.14 Simplified Report Sample #14

This medical report is talking about some pictures that were taken of the inside of a person's body. The pictures show how the person's body is doing and how it has changed since the last time they got pictures taken.

The pictures show that the person's heart is normal and there is no extra fluid around it. The person's thyroid gland is bigger than usual, but there is no extra fluid in their chest and they do not have an infection in their lungs. The person has some hard lumps on the inside of their chest that have not changed. They

also have some spots on their lungs that were caused by cancer. These spots have gotten smaller and have some holes in them, which is a good sign that the person is getting better.

The pictures also show that the person's liver looks normal and there are no cancer spots on it. The person's gallbladder has a small stone, but it is not causing any problems. The person's other organs, like the spleen and pancreas, look normal. The person's kidneys and urinary system are also normal and there is no extra fluid in the person's belly. The person had surgery to remove some cancer spots in their intestine and there are no new cancer spots there. However, there are some hard lumps on the person's back that doctors will keep an eye on.

Finally, the pictures do not show any new bones with cancer or any broken bones. Overall, the person's body is showing a good response to treatment and the cancer is getting better in some areas.

B.3.15 Simplified Report Sample #15

This medical report is about a person who has cancer that has spread from their colon to other parts of their body. The doctors used special pictures called CT scans to see inside the person's body. They looked at the person's chest and saw that their heart is normal and not causing any problems. They also saw some changes in the cancer in the person's lungs that suggest the treatment the person is receiving is working. The doctors also looked at the person's abdomen and saw that the cancer has not spread to the liver or other organs there. They saw some changes in the lymph nodes in the person's belly that may need to be watched closely. Finally, the doctors looked at the person's bones and did not see any signs of cancer or broken bones. Overall, it looks like the treatment the person is receiving is helping to slow down the cancer.

C Questionnaire

C.1 Questionnaire Design

Questionnaire - Quality of Simplified Radiological Reports

Date: 21.12.2022

Consent

I consent to participate in this anonymized questionnaire and to the publication of my answers.

- ◯ yes
- ◯ no

Experience

How many years of experience do you have as a radiologist (starting from your first year of residency)?

What this questionnaire is about

Based on this questionnaire we will assess the quality of radiological reports that have been simplified by the machine learning language model ChatGPT.

You will be presented 3 pairs of radiological reports. Each pair consists of (a) an original radiological report and (b) a respective simplified version, created with ChatGPT.

We kindly ask you to read all reports carefully and answer the questions of this questionnaire.

How to answer this questionnaire

The questions can be found after each pair of reports ((a) original radiological report + (b) simplified report).

Give your answers to the questions by ticking a number on the Likert scale and by writing your answer in the space provided.

If you want to correct your answer, cross out your wrong answer clearly. On the Likert scale, also tick a new number.

Who created this questionnaire

This questionnaire was jointly created by: Jakob Dexl, Michael Ingrisch, Katharina Jeblick, Andreas Mittermeier, Bastian Sabel, Balthasar Schachtner, Johanna Topalis, Tobias Weber, Philipp Wesp.

Department of Radiology, University Hospital, LMU Munich.

Figure C1.1: Meta questions and cover for the questionnaire.

Questions on reports #1

1. The simplified radiological report is factually correct.

1	2	3	4	5
Strongly agree	Agree	Neutral	Disagree	Strongly disagree

   (a) Highlight all incorrect text passages (if applicable) of the simplified report with a text marker.

2. Relevant medical information for the patient is included in the simplified radiological report.

1	2	3	4	5
Strongly agree	Agree	Neutral	Disagree	Strongly disagree

   (a) List all relevant medical information, which is missing in the simplified report (if applicable).

3. The simplified report leads patients to draw wrong conclusions, which might result in physical and/or psychological harm.

1	2	3	4	5
Strongly agree	Agree	Neutral	Disagree	Strongly disagree

   (a) List all potentially harmful conclusions, which might be drawn from the simplified report (if applicable).

Figure C1.2: Questions concerning one of the simplified reports provided to the radiologist in the questionnaire.

C.2 Answers to the Questionnaire

Table C.2: Answers of the radiologists in the questionnaires. The answers to question 1a (incorrect text passages) are highlighted in the simplified reports in Appendix B. Exper. = Experience

Participant	Exper.	Sample	Q1	Q2	Q2a	Q3	Q3a
1	4	B.1.1	1	1	-	4	-
		B.2.1	3	2	-	2	Contradicting results (new brain lesion vs. no new brain lesion)
		B.3.1	1	1	-	5	-
2	1	B.1.2	2	1	-	4	-
		B.2.2	2	2	no intermediate or recent ischemia	4	Glioblastoma in conclusion, too specific<br>it's a DD not yet diagnosed.
		B.3.2	2	1	-	4	-
3	0.5	B.1.3	2	2	-	4	-
		B.2.3	2	3	-	3	Significant growth (almost doubled) should maybe not be played down with the word "small" growth and I would also use the word significant in the conclusion.
		B.3.3	2	2	-	3	I would leave out the conclusion that it "is a good sign and means that a person is responding to the treatment" or move it to the last paragraph of the report.
4	3.5	B.1.4	1	2	-	4	-
		B.2.4	2	4	In conclusion growth of the lesion parietooccipital is missing	3	Information in conclusion about the lesion showing growth is missing.
		B.3.4	2	2	-	4	-
5	6	B.1.5	2	1	Cartilage damage is on the inner side of the knee (thigh bone)	5	-
		B.2.5	1	1	-	3	-
		B.3.5	4	2	Spot vs metastasis<br>decreasing solid portions in pulmonal metastasis	2	"the parts that are staying the same size are changing" unclear if good or bad<br>not clear that spots are pulmonal metastases
6	5	B.1.6	2	2	Infraction lateral tibia plateau	4	-
		B.2.6	2	2	-	3	DD ≠ "is"
		B.3.6	2	2	-	4	-
7	10	B.1.7	2	2	-	4	-
		B.2.7	2	2	Second/ distant manifestation of known GBM in right frontal lobe which has been resected	3	GBM is one (likely) DD which implies that other DDs exist (e.g. radionecrosis). This uncertainty is not conveyed in the simplified report
		B.3.7	2	1	-	4	-
8	0.4	B.1.8	3	2	-	3	-
		B.2.8	4	2	-	3	lacking communication that it might be another (not GBM) brain tumor and weighing of possibilities

		B.3.8	4	4	-	2	Lacking communication about the limitations of CT and the factual relevance of size decrease of metastases.
9	4	B.1.9	2	1	-	4	-
		B.2.9	2	2	-	2	brain not damaged!
		B.3.9	4	2	-	2	lymph nodes
10	5	B.1.10	2	1	-	4	-
		B.2.10	2	2	-	4	-
		B.3.10	2	2	-	4	-
11	11	B.1.11	2	2	-	3	-
		B.2.11	2	2	Localisation of the present tumor versus localisation of the excised tumor.	2	Misunderstanding which lesion is stable and which one is in progress can lead the patient to some wrong expectations.
		B.3.11	1	1	-	4	-
12	0.5	B.1.12	2	2	-	4	-
		B.2.12	2	2	-	3	-
		B.3.12	3	2	-	3	-
13	22	B.1.13	2	2	-	4	-
		B.2.13	4	3	-	2	Changes happened recently (how recently?)<br>Some extra fluid ≠ sign. incr. edema
		B.3.13	4	2	not thyroid infection but enlargement<br>there is spread of cancer → lungs	2	there is no (new) spread of cancer
14	8	B.1.14	1	1	-	5	-
		B.2.14	1	1	-	5	-
		B.3.14	3	2	Back is not specific enough or wrong as the retroperitoneum is actually affected	2	Patient might be misled to look at cancer spots on their back.
15	10	B.1.15	2	2	report is about the knee	4	-
		B.2.15	1	1	-	5	-
		B.3.15	1	1	-	5	-
